# LEOD-Net: Learning Line-Encoded Bounding Boxes for Real-Time Object Detection

**DOI:** 10.3390/s22103699

**Published:** 2022-05-12

**Authors:** Hatem Ibrahem, Ahmed Salem, Hyun-Soo Kang

**Affiliations:** 1 Department of Information and Communication Engineering, School of Electrical and Computer Engineering, Chungbuk National University, Cheongju-si 28644, Korea; hatem@cbnu.ac.kr (H.I.); ahmeddiefy@cbnu.ac.kr (A.S.); 2 Electrical Engineering Department, Faculty of Engineering, Assiut University, Assiut 71515, Egypt

**Keywords:** object detection, convolutional neural networks, line detection, real-time processing

## Abstract

This paper proposes a learnable line encoding technique for bounding boxes commonly used in the object detection task. A bounding box is simply encoded using two main points: the top-left corner and the bottom-right corner of the bounding box; then, a lightweight convolutional neural network (CNN) is employed to learn the lines and propose high-resolution line masks for each category of classes using a pixel-shuffle operation. Post-processing is applied to the predicted line masks to filtrate them and estimate clear lines based on a progressive probabilistic Hough transform. The proposed method was trained and evaluated on two common object detection benchmarks: Pascal VOC2007 and MS-COCO2017. The proposed model attains high mean average precision (mAP) values (78.8% for VOC2007 and 48.1% for COCO2017) while processing each frame in a few milliseconds (37 ms for PASCAL VOC and 47 ms for COCO). The strength of the proposed method lies in its simplicity and ease of implementation unlike the recent state-of-the-art methods in object detection, which include complex processing pipelines.

## 1. Introduction

**Figure 1:**
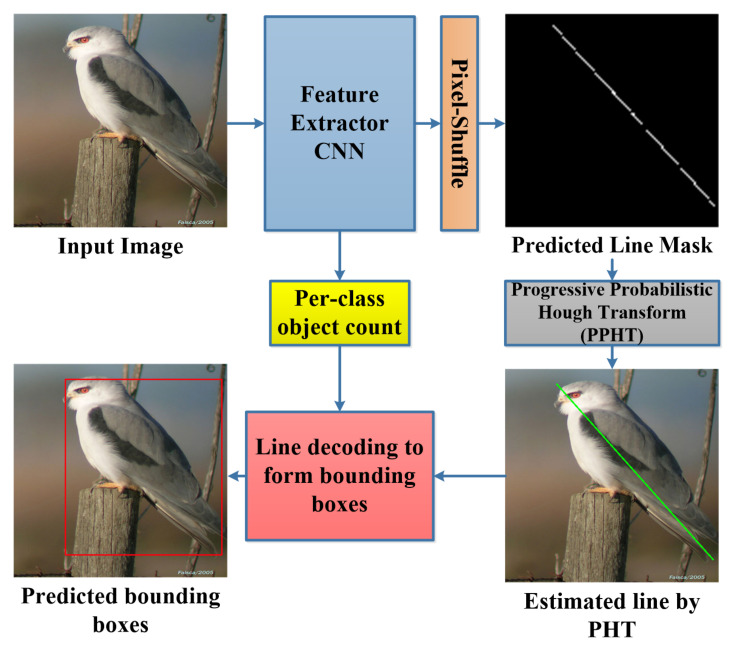
Overview of the proposed object detection method using line-encoded bounding boxes.

Object detection tasks are one of the most important tasks in computer vision as it is mainly included in understanding and analyzing a scene in images, and this task becomes more efficient when it is performed in real time in order to be useful in live-video processing. The object detection is usually performed using bounding box regression by predicting the x and y values of the top-left corner in addition to the width and the height of the box. The recent object detection methods are mainly classified into two main categories: two-stage methods and single-stage methods. The two-stage method is usually complex as it consists of a stage for object proposals and another stage for object classification and bounding box regression; this concept is applied in many recent methods such as RCNN [1], Fast RCNN [2], Faster RCNN [3], and Mask RCNN [4]. The two-stage methods attain high mean average precision (mAP); however, they are extremely slow (0.2–10 frames per second (FPS)) as such pipelines are computationally expensive and include complex processing techniques. On the other hand, the single-stage object detection methods employ fully convolutional neural network architectures and perform the object detection task at a high speed (20–140 FPS) such as Yolo V1 [5], V2 [6], V3 [7], V4 [8], Single Shot Detection (SSD) [9], and RetinaNet [10]; however, the mAP values for such methods are lower than those for the two-stage methods as they mainly depend on small-scale grids that introduce accuracy loss in learning the bounding box coordinates. A good object detection method should have a trade-off between high accuracy and high processing speed, which is the goal of this paper—achieving a relatively high speed and accuracy. An overview of the proposed method is shown in Figure 1.

We propose a fast object detection method by training a CNN model to predict line-encoded bounding box masks for each class object. The CNN predicts high-resolution line masks using a lightweight pixel-shuffle operation [11] inspired by a technique employed in the image super-resolution task. An important post-processing stage is employed to filter out the predicted lines and to estimate fine bounding boxes out of the lines by exploiting the progressive probabilistic Hough transform (PPHT) [12] to find clear lines based on a proposed iterative technique under constraints. The contribution of the work presented is as follows:

- We propose new bounding box encoding and learning techniques. The bounding box encoding technique is based on encoding the top-left and bottom-right corners of the bounding box in a single line learnable by segmentation map prediction.
- We propose a robust post-processing technique to solve the problem of multiple detections of the same object and the problem of many detections of a deformed line of a single object.
- The proposed method can successfully achieve a good trade-off between speed and accuracy. It realizes real-time processing (27fps) while keeping a high mAP in object detection.

The rest of the paper is organized as follows: Related work, which details the recent methods in object detection; the Proposed Method, which contains the details of our implementation; Benchmarks For Training and Validation, which contains the dataset employed to train and test our proposed method; Evaluation Metrics of Object Detection and Ablation Study, which contains two main studies on the scale of the line mask and the up-sampling techniques employed in our method; Complexity Analysis of the Proposed Model, Experimental Results, Limitation, and Future Work, and finally the conclusion of the paper.

## 2. Related Work

The recent deep-learning-based object detection methods have shown a superior ability of the CNN models to learn and perform object detection accurately and rapidly. As we previously mentioned in Section 1, there are two main CNN-based methods for object detection: two-stage methods and single-stage methods. Sermanet et al. [13] proposed Overfeat, which is one of the early deep-learning-based two-stage object detection methods in which they trained a CNN image classifier (AlexNet [14]) and then applied the trained classifier on every batch of the image using a sliding window with different window scales; however, this method was very slow due to the high number of computations required for classification of each image patch. The authors of [1] proposed RCNN, which is a two-stage CNN model for object detection and employed a selective search method [15] to propose a limited number of regions (typically 2000 regions) for classification by a CNN image classifier (VGG16 [16]) instead of the classification of the whole image with different scale windows; this method still provides an extremely low frame rate (0.2 FPS). Later in 2015, Girshick et al. [2] proposed Fast R-CNN in which the author reduced the complexity of RCNN by feeding the image to a CNN (VGG16) then applying the selective search method on the feature maps obtained from the CNN instead of applying it on the whole image; the author also proposed ROI pooling to reshape all the proposed features into squares and then feeding them to a class classification + bounding box-regression CNN. Fast R-CNN attained a relatively low speed of 2 FPS, although much better (10× faster) than RCNN. Ren et al. [3] proposed Faster R-CNN in which they solved the drawbacks in both R-CNN and Fast R-CNN by eliminating the need for the computationally expensive selective search method; they feed the input image to a CNN (VGG16) to propose a few regions (typically 300 regions) for classification and then another CNN (VGG16 or ResNet [17]) is used to classify the regions and regress the bounding boxes. Faster R-CNN attained a high mAP at a speed of 10 FPS, which is still relatively low.

The recent single-stage object detection methods showed an average accuracy but attained a high speed in frame processing. The first single-stage object detection method was proposed by [5] under the name “You Only Look Once” or YOLO; they proposed a grid-based detection method using a convolutional architecture (specifically Darknet) in which each cell in the grid predicts the class category in that cell in addition to x, y, w, and h coordinates of the bounding box where x and y are the coordinates of the top-left corner of the cell and w and h are the widths and the height of the bounding box of the object exist in that cell; however, although YOLO is fast enough for real-time processing (it can work with a speed of 45 FPS), it has a major problem, which is the failure to detect small objects, as the grid was too small (7×7). YOLOV2 or YOLO9000 [6] was proposed by the first and the last authors of YOLO to improve the speed and the accuracy of YOLO; they added patch normalization layers after the convolutional layers in the YOLO architecture, which improved the mAP by 2%; they also used bigger image size, typically 448×448, instead of the small image size (224×224) used in the initial YOLO version; this modification also increased the mAP by 4%. They also reduced the original Darknet architecture from 26 layers to 19 layers (Darknet-19) to speed up the process (they achieved a frame rate of 67 FPS at 448×448 image size). They also proposed anchor boxes to limit the shapes of the predicted bounding boxes to specific object-based shapes instead of the arbitrary boxes predicted by YOLO. YOLOV3 is proposed by [7] of YOLOV2 to improve the detection of small objects; the authors employed Darknet-54, which is a deeper CNN than YOLOV1 and V2 and also employed multiple-scale detection using an architecture similar to the feature pyramid network (FPN) [18]. The detection in YOLOV3 is achieved at three different scales (small, medium, and large) and a non-maximum suppression is applied to obtain the detections with the highest scores. YOLOV3 attained a higher mAP than YOLOV1 and V2 but the frame rate was reduced to 35 FPS at 416×416 image size. YOLOV4 was proposed by [8], where they improved the mAP by 10% over YOLOV3 by presenting a new backbone (CSPDarknet53) employing cross-spatial partial connections. They proposed three main parts: backbone, neck (path aggregation networks [19] with spatial pyramid pooling [20]), and head (dense prediction block); YOLOV4 attains a speed of 62 FPS at the best mAP value with an image size of 416×416. Duan et al. [21] proposed Center-Net, a keypoint-based method to detect the objects using three points (top-left, center, and bottom-right points) and achieved high accuracy in detection. Tan et al. [22] proposed EfficientDet, which is a fast and accurate object detection method based on the successful architectures of EfficientNet [23] originally proposed for classification. The author also proposed the bi-directional feature pyramid network (BiFPN), which allows the feature fusion of multiscale features.

In the proposed method we employ a CNN backbone (specifically Xception [24]) to extract the image features, then the obtained low scale features are upscaled using the pixel-shuffle algorithm inspired by the efficient sub-pixel CNN [11] originally presented for the real-time image super-resolution task. This algorithm can up-scale many low-resolution images of shape $W\times H\times r^{2}C$ (where $r^{2}$ is the scaling factor) into a high-resolution image of shape (rW×rh×C) through pixel shuffling from the depth channel. This algorithm is fast and efficient in the construction of higher resolution images and especially segmentation masks as explored in detail in our previous research [25,26]. The progressive probabilistic Hough transform (PPHT) [12] is a popular method for straight line detection from a small set of edge points instead of all edge points used in the standard Hough transform (SHT) [27], thus PPHT is much faster than HT. As PPHT is an iterative method, a random edge point is selected for each iteration for voting, then the condition of the line is tested. If a specific line has a large number of votes from the randomly selected points, the stopping rule is satisfied and the line is approved as a detection. PPHT can be tuned using the algorithm parameters to control the estimated line/lines, such as controlling whether to combine multiple sparse points or not based on their alignment. The line estimation using PHT is efficient and rapid enough to be performed as a post-processing step to the detected lines in our proposed method, which targets the real-time object detection task.

## 3. Proposed Method

The proposed method consists of three main parts. Firstly, the backbone used for feature extraction (Xception-16) is a modified version of Xception with two output branches. Secondly, we use the pixel-shuffle operation, which is used to upscale the final features based on the depth channel. Finally, the post-processing stage combines the probabilistic Hough transform and the per-class object count to decode the line and obtain the bounding boxes.

### 3.1. Xception-16 Architecture

**Figure 2:**
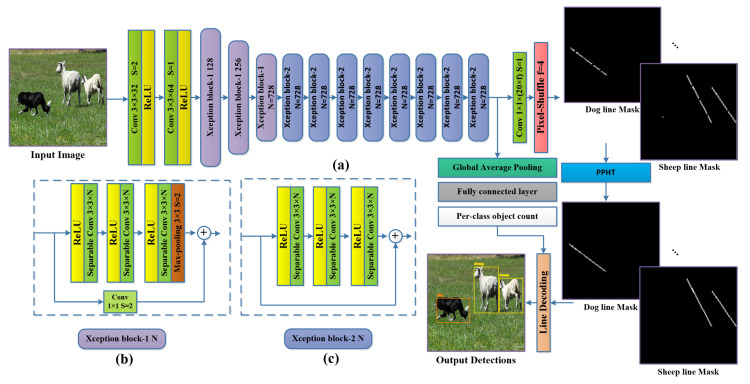
The proposed method. (**a**) The Xception-16 architecture with two branches: one for predicting the line mask for each object and the other branch for per-class object count by regression, which is employed in the post-processing stage of the line decoding. (**b**) Xception block-1 is the Xception block that consists of three sequential RELU+ 3×3×N separable convolution2D and a 3 × 3 max-pooling with stride (s) of 2, a skip connection with 1×1 convolution2D and stride of 2. (**c**) Xception block-2 is the Xception block that consists of three sequential RELU+ 3×3×N separable convolution2D.

Xception [24] is an efficient feature extractor network presented initially for ImageNet ILSVRC [28] image classification and attained a top-5 accuracy of 0.945, which is relatively high compared to the current state-of-the-art (SOTA) methods. Chollet et al. [24] proposed the depth-wise separable convolution (DW-Conv) as the building block of Xception architecture. DW-Conv consists of two convolution operations. Firstly, the depth-wise convolution performs convolution on each channel separately. Secondly, the point-wise convolution applies a 1×1 convolution on the input. DW-Conv is much faster than the standard convolution as it learns fewer parameters so it is key to the fast processing in our proposed method. Xception also proved to be a good feature extractor in recent research for multiple computer vision tasks, as it proved to be light enough for real-time applications because of the relatively low FLOPs count and a number of other parameters [29,30]; it also proved to be compatible with the pixel-shuffle [11] operation (also employed in our proposed method and is introduced in Section 3.2) as Xception with the pixel-shuffle showed high accuracy in performing the semantic segmentation task in DTS-Net [25]. As our method performs the semantic segmentation as a secondary task to predict the encoded line, we adopted a modified version of Xception for its robustness and high accuracy. We propose Xception-16, which cuts the original Xception architecture at the layer `block13_sepconv2_act’, which is equivalent to the input image scale divided by 16 (i.e., input image of size 448×448 produces features of scale 28×28 using the proposed Xception-16), then we add two branches, the first with a convolution2D layer followed by the pixel-shuffle operation to construct the line mask in the required scale and the second branch with global average pooling (GAP) followed by a fully connected layer (FC) to predict the per-class object count. The name Xception-16 comes from the final feature scale or the down-scaling obtained from the network, which is 1/16 of the input image size. The Xception-16 architecture is shown in Figure 2a.

### 3.2. Pixel-Shuffle as a Feature Map Upscaling Algorithm

**Figure 3:**
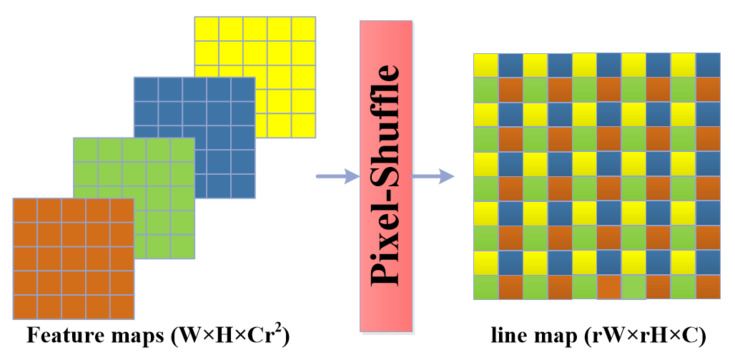
Pixel-shuffle: the pixels are mapped from the small-scale feature maps of size ($W\times H\times Cr^{2}$) to form super pixels in the large-scale line map of the size (rW×rH×C).

The pixel-shuffle algorithm is proposed by [11] for real-time image super resolution, as the algorithm is fast and efficient in constructing large-scale images from many small-scale images through pixel-reordering from each small-scale image to form super pixels in the large-scale images, as shown in Figure 3.

The pixel-shuffle algorithm can upscale small-scale images of shape $W\times H\times Cr^{2}$ into a large image of scale rW×rH×C through a rearranging operation to map the pixels depending on the location of each pixel according to (1):

(1) L(x,y,C)=S(x/r,y/r,C.r.mod(y,r)+C.mod(x,r)+C)

where *L* and *S* are the large-scale and small-scale images, *x* and *y* are the horizontal and the vertical location of a pixel, *C* is the number of channels, *r* is the square root of the upscaling factor $r^{2}$, and *mod*() is the modulus operation. In our proposed method, we add a $1\times 1\times Nr^{2}$ convolutional2D layer after the Xception-16’s last layer, which produces a 728 feature map of 1/16 of the input image size to adjust the depth channels so that after the pixel-shuffle, it produces line maps equivalent to the number of the categories at the required scale. We try different upscaling factors to obtain 1/1, 1/2, 1/4, and 1/8 of the input image size in the experiment section to reveal the effect of the line-map scale on the mean average precision. The objective function used for line segmentation is a pixel-wise multilabel classification to allow the existence of multiple lines in the same location but in different masks; the employed function is binary cross-entropy as shown in Equation (2):

(2) $$SegLoss=-\frac{1}{CP}\sum_{i=0}^{C}\sum_{j=0}^{P}y_{i,j}*log\left(\hat{y_{i,j}}\right)+\left(1-y_{i,j}\right)*log\left(1-\hat{y_{i,j}}\right)$$

where *C* is the number of classes, *P* is the number of pixels in the line mask, *y* is the ground truth image label, and $\hat{y}$ is the predicted image label.

### 3.3. Per-Class Object Count Regression

The second output branch from the proposed CNN is used to predict the object count of each class. The object count is used to ensure that the number of the detected objects equals the predicted number of objects per class. In the case of non-matching, a correction technique is followed using pre-defined PPHT parameter cases. The prediction of the per-class object count has to be performed by applying the GAP layer to the output from the Xception-16 backbone to obtain a 1D feature vector, then an FC layer is added to obtain dense predictions of the objects per class. This task is performed through regression with a mean squared error loss as shown in Equation (3):

(3) $$RegLoss=\frac{1}{N}\sum_{i=0}^{N}{\left(y_{i}-\hat{y_{i}}\right)}^{2}$$

where *y* and $\hat{y}$ are the ground truth and the predicted object count, respectively, and N is the number of classes. The overall loss is the sum of the two losses in (2) and (3) with equal weights as shown in (4).

(4) Overall_Loss=SegLoss+RegLoss

### 3.4. Bounding Box Encoding and Line Decoding Algorithms

**Figure 4:**
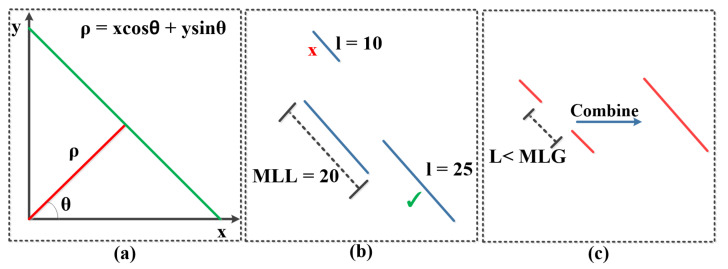
PPHT algorithm parameters illustration. (**a**) ρ and θ of a line in polar coordinates. (**b**) Minimum line length condition to accept or reject lines. (**c**) Maximum line gap (MLG) condition to combine two points or line segments.

The PPHT [27] algorithm contains five main parameters that should be carefully tuned in order to achieve the best line detection results. There are three parameters related to the edge points accumulator or line detector (ρ, θ, and *t* where ρ is the resolution of the distance of the accumulator in pixels, θ is the resolution of the angles of the accumulator in radians, and *t* is the votes threshold of the accumulator to verify any line detection). Another two parameters, which are the minimum line length (MLL) and the maximum line gap (MLG), are used to define the shortest length of the line to be considered as detection and the maximum gap between any two points to consider them as one line, respectively. A visual illustration of PPHT parameters is shown in Figure 4.

In the proposed method, the Xception-16 network is trained to predict line masks using a binary segmentation approach. The ground truth lines are generated from the bounding box annotations provided in each dataset used. Each line is produced depending on each object, whereas the line beginning is the top-left corner and the end of the line is the bottom-right corner of the bounding box. Formally, all the bounding boxes are encoded in a negative-slope line format. Since the objects are encoded in a one-pixel-thick line, that means that each line is unique and easy to be separated from other lines of the same class as there is a line mask for each class category and the lines have different slopes according to the alignment of the objects, which are different for the instances of the same class. We apply PPHT to the predicted lines for each class category obtained from the Xception16+Pixel-shuffle, but the PPHT algorithm can produce a different number of lines based on the selection of the parameters. As such, we use the per-class object count prediction to provide a reference of the number of the lines that should be produced from each line mask of the classes. If the algorithm fails to match the exact per-class object count, it performs distance measurements between the produced lines from PPHT in each case and the count vector to predict, as much as possible, lines close to the true number of lines in the count vector. This proposed algorithm for line detection depends mainly on the scale of the line masks; as such, we apply PPHT three times (three parameter sets are empirically selected to detect small, medium, and large objects) on the masks that have line segments; each time we try three pre-selected different θ resolution, ρ resolution, and threshold (*t*) but we keep the MLL and MLG at fixed values dependent on the line mask scale. The exact values selected for each scale are described in greater detail in the Experimental Results section. When the number of the detected lines per class (using PPHT with the designed conditions) matches or closely match the number of the objects per class (obtained from the per-class object count predictions of Xception-16), the bounding boxes are formed based on the detected lines by decoding the beginning and the end of the line to obtain the top-left and the bottom-right corners of the bounding boxes. The sequence of the algorithm is stated in Algorithm 1. In Algorithm 1, the decode_boxes() function refers to the decoding of the begging and the end of detected lines to the top-left and the bottom-right of the bounding boxes; show_boxes() and show_lines() are just drawing functions for visualization of the detected lines and bounding boxes.
**Algorithm 1** Line Decoding**Input:** predicted line-masks LM, number of classes *N*, Predicted Count vector *C*Initialize cases=3, lines=[], boxes=[], $\theta =[\theta_{1},\theta_{2},\theta_{3}]$, $l=[l_{1},l_{2},l_{3}]$, $d=[d_{1},d_{2},d_{3}]$, diff= 0**for **i=0** to*** N*** do**    **for** j=0 **to** cases **do**        **if** case==j **then**           $line_{i}$ = PPHT(LM, ρ = 1, θ = $\pi /\theta_{j}$, $t=t_{j}$, $MLL=l_{j}$, $MLG=d_{j}$)           lines.append($line_{i}$)           diff= 100           **for** K=0 **to **len(lines) **do**               **if** abs($C_{i}$—len(lines)) < diff **then**                   diff = abs($C_{i}$—len(lines)) < diff                   lines = lines[0:$C_{i}$]               **end if**           **end for**           boxes.append(decode_line($line_{i}$))        **end if**    **end for**    show_boxes(boxes), show_lines(lines)**end for**

## 4. Benchmarks for Training and Validation

For the proposed method training and validation, we employ three common object detection datasets: PASCAL VOC2007 [31], VOC2012 [32], and MS-COCO2017 [33]. PASCAL VOC2007 is a popular dataset for the common objects in the scenes: it consists of 20 classes of objects. The dataset contains 5,011 images for training and 4,952 images for validation. PASCAL VOC2012 has the same classes as PASCAL VOC2007 but with different training and validation images: it consists of 5716 training images and 5823 images for validation. For better model training, we trained the proposed model on both PASCAL VOC2007 and VOC2012 training sets and we tested the model on the PASCAL VOC2007 dataset test set. For training and testing on PASCAL VOC datasets, we used an image size of 448×448. The third dataset used for validation was MS-COCO which is a larger dataset for common objects in the scenes and contains 80 class categories. The MS-COCO dataset consists of 118,287 training images and 5000 validation images. We used an image size of 560×560 for training and testing on the MS-COCO dataset. The bounding box annotations are provided for the three mentioned datasets.

## 5. Evaluation Metrics of Object Detection

For the proposed method of performance measurement, we evaluated the proposed method on PASCAL VOC2007, VOC2012, and COCO minival (validation set of MS-COCO2017) to measure the mean average precision (mAP) at an intersection over union greater than a threshold; 0.5 is used as a threshold in the evaluation of most object detection methods. The average precision (AP) metric is the measure of the average value of the precision for recall values over 0 to 1. The precision and the recall can be defined as in Equation (5):

(5) $$\mathrm{Precision}=\frac{\mathrm{TP}}{\mathrm{TP}+\mathrm{FP}},\;\mathrm{Recall}=\frac{\mathrm{TP}}{\mathrm{TP}+\mathrm{FN}}$$

where TP, FP, and FN are the true positive, false positive, and false negative of the predictions, respectively. The precision measures how accurate the predictions are and the recall measures whether the model can predict the positives. The AP is the area under the precision-recall curve. The mAP is the mean value of the AP over all the classes; it is usually measured at an IOU value of 0.5, but in MS-COCO evaluation several IOU values are used (from 0.5 to 0.95 with step of 0.05) and the average of those IOU values is calculated to obtain AP${}_{box}$. Further, the AP for the small, medium, and large objects is calculated according to the annotation of the objects in the image. IOU can be defined as in Equation (6):

(6) $$\mathrm{IOU}=\frac{Bbox_{pred}\cap Bbox_{gt}}{Bbox_{pred}\cup Bbox_{gt}}$$

where $Box_{pred}$ and $Box_{gt}$ are the predicted and the ground truth bounding boxes, respectively.

## 6. Training and Test Setup

The proposed method was trained using a desktop computer with Nvidia RTX3090 GPU, Intel Corei7-8700 CPU @3.20 GHz clock speed, and 64 gigabytes of RAM. The training was performed using the Tensorflow Keras environment where the trained models have been trained using Adam’s optimization method with an initial learning rate of 0.001 for approximately 250 epochs. A translation and horizontal flipping operations are adopted during training as an augmentation to prevent overfitting and provide more generalization of data. The original Xception model is initialized with ImageNet classification weights then the network is cut to be the modified version “Xception-16” to speed up the training process. The inference was performed using an Nvidia Titan XP GPU with the other configuration mentioned before.

## 7. Ablation Study

We performed two main studies: one on the scale of the line mask used for box decoding and the second on the up-sampling techniques used for forming the line mask.

### 7.1. Study on the Scale of the Line Mask

**Figure 5:**
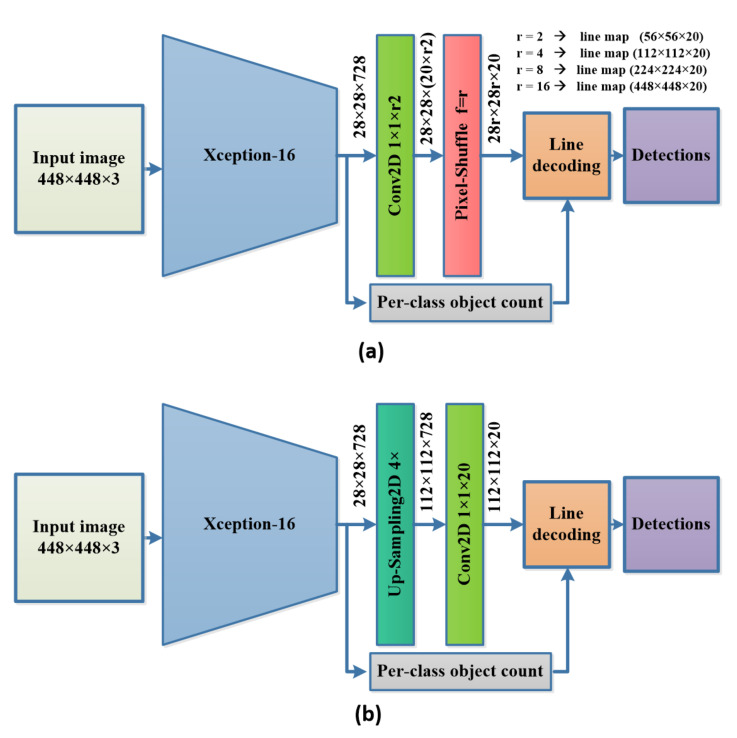
Ablation study architectures. (**a**) The architecure for the different scales of the line mask (1/8, 1/4, 1/2, 1/1 of the input image). (**b**) The architecure for the different upsampling techniques (bilinear, nearest neigbour, pixel-shuffle).

We performed four separate training experiments for the proposed model using four different up-sampling scales of the pixel-shuffle module. We experimented with 1/8, 1/4, 1/2, and full scales to determine which scale has the best performance in terms of mAP on PASCAL VOC2007. The number of channels at the final Conv2D layer before the pixel-shuffle is changed so that the obtained line masks are formed at the desired scale. The value of r of the pixel shuffle is also changed according to that too (r = 2 for 1/8 scale, r = 4 for 1/4 scale, r = 8 for 1/2 scale, and r = 16 for full-scale) as shown in Figure 5a.

**Figure 6:**
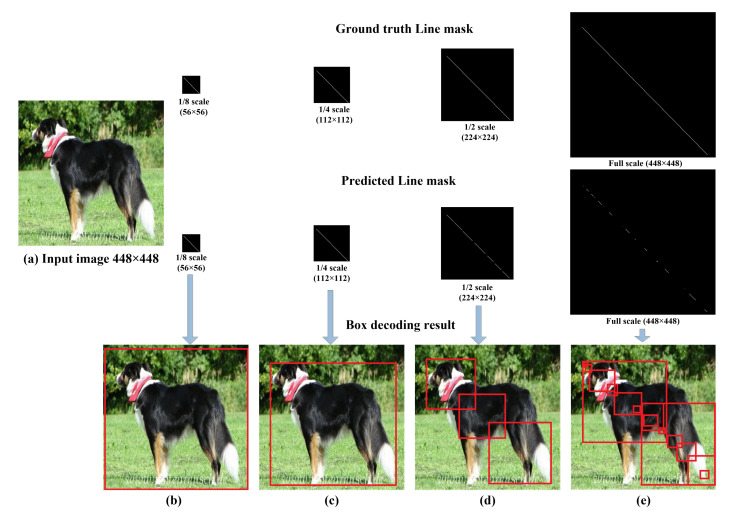
Comparison between the decoded boxes using different scales of the line masks during training and test. (**a**–**e**) show the input image and the decoded boxes using the predicted line masks of scales 1/8, 1/4, 1/2, and 1, respectively.

Figure 6 shows the obtained line mask in a sample test image with the corresponding decoded bounding box in each case. The smallest scale (1/8 scale) gives a solid-continuous line in the line mask but due to the small scale, the decoded box using PPHT is too wide and does not exactly fit the object. In the case of the 1/4 scale, the detected line has a few small gaps but still, the PPHT is able to detect the line and merge the line segments. In the case of the 1/2 scale, the PPHT detects multiple segments and cannot easily merge the line segments and fails in many cases, resulting in a big loss in the mAP; the full-scale case also generates many tiny line segments and sparse points, which totally confuses the PPHT algorithms and make it impossible to detect the objects properly. The bad line mask results in larger scales and comes from the fact that the density of the line pixels is very small compared to the scale of the mask as shown in Table 1. As a result of the previous experiments, we selected the 1/4 scale, which has a good trade between the tightness of the bounding box and the relatively high density of line pixels, which is enough for PPHT to detect it without much effort in tuning the PPHT algorithm parameters. The best parameters of PPHT (shown in Table 1) were tuned manually by trial and error to obtain the best possible mAP. We also compared the speed of the model on different scales and, as expected, the lower the scale, the higher the frame rate; we selected the 1/4 scale as the best one based on the mAP sacrificing the better frame rates in the case of the 1/8 scale.

### 7.2. Study on the Up-Sampling Technique for the Line Mask

We experimented with three different up-sampling techniques to form the line masks. The up-sampling techniques up-sample the final features extracted using Xception-16 four times, and then a Conv2D layer is used to reshape the number of the filters to be equal to the number of classes, as shown in Figure 5b. We trained each model using each one of the up-sampling techniques and then compared the pixel-shuffle with the bilinear and nearest neighbor up-sampling techniques at a line mask scale of 1/4 of the input image.

**Figure 7:**
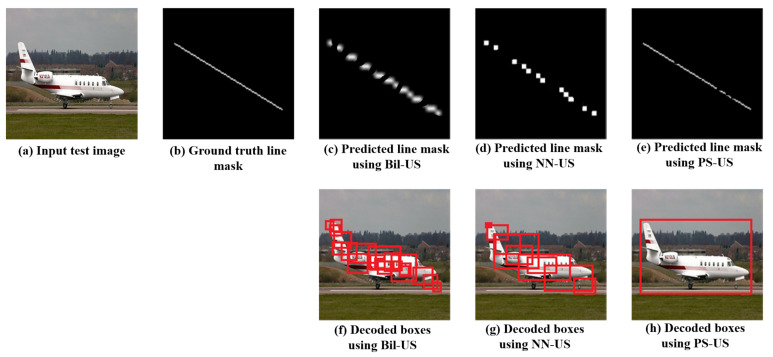
Comparison between the predicted line mask and the decoded boxes in case of using the bilinear up-sampling (Bil-US), nearest neighbor up-sampling (NN-US), and pixel-shuffle up-sampling (PS-US).

The bilinear up-sampling and the nearest neighbor up-sampling showed a poor performance in obtaining solid lines; they produced many gaps and thick line segments, which result in low mAP values (lower than 10) so we could not produce notable results to compare with the pixel-shuffle-based results. In spite of our effort to tune the PPHT parameters, the performances of the bilinear and the nearest-neighbor-based up-sampling are much lower than that for the pixel-shuffle, which produces a thin solid line with few gaps, as indicated in the sample results shown in Figure 7.

## 8. Complexity Analysis of the Proposed Model

We analyze the proposed model including the Xception-16 feature architecture plus the two branches (The pixel-shuffle for line segmentation with 1/4 scale of the input image and the fully connected layer for the per-class regression). The analysis was performed using the TensorFlow profiler [34], specifically the tf.profiler.ProfileOptionBuilder.float_operation() function, to calculate the number of floating point operations (FLOPs) for the different layers, i.e., Convolution2D, Depthwise separable convolution2D, and max pooling2D, and the other operations in the model, i.e., multiplication and addition operations. Table 2 shows a detailed analysis of the proposed CNN models with the two image sizes used for PASCAL VOC and MS-COCO datasets. It is obvious in Table 2 that the convolution2D operations take most of the computations and the depth-wise separable convolution takes fewer computations since the depthwise separable convolution is much less complex than the conventional convolution2D operation.

## 9. Experimental Results

The proposed method was trained to predict line masks with a scale of 1/4 of the input image, i.e., for PASCAL VOC2007 and VOC2012, the input RGB images are resized to 448×448 and the ground truth line masks (which are binary masks) are made at 112×112 and, for MS-COCO, we used a bigger image size of 560×560 and the line masks have the size of 140×140.

### 9.1. Evaluation of the Per-Class Count Regression

We evaluated the branch of the per-class object counts separately to ensure the ability of the model to predict the number of objects of each class in the image. The obtained values in the predicted count vector are floating numbers; we round the vector values to the nearest integer values first and then we measure the accuracy of predicting the integer value of each object. We could attain a counting accuracy of 97% on the PASCAL VOC2007 test set and of 92% on the MS-COCO minival (MS-COCO val2017). Those accuracies are the basis of the success of our method, as in our algorithm, we force the PPHT to predict lines equivalent to the per-class object count.

### 9.2. Evaluation Results on Pascal Voc2007

**Figure 8:**
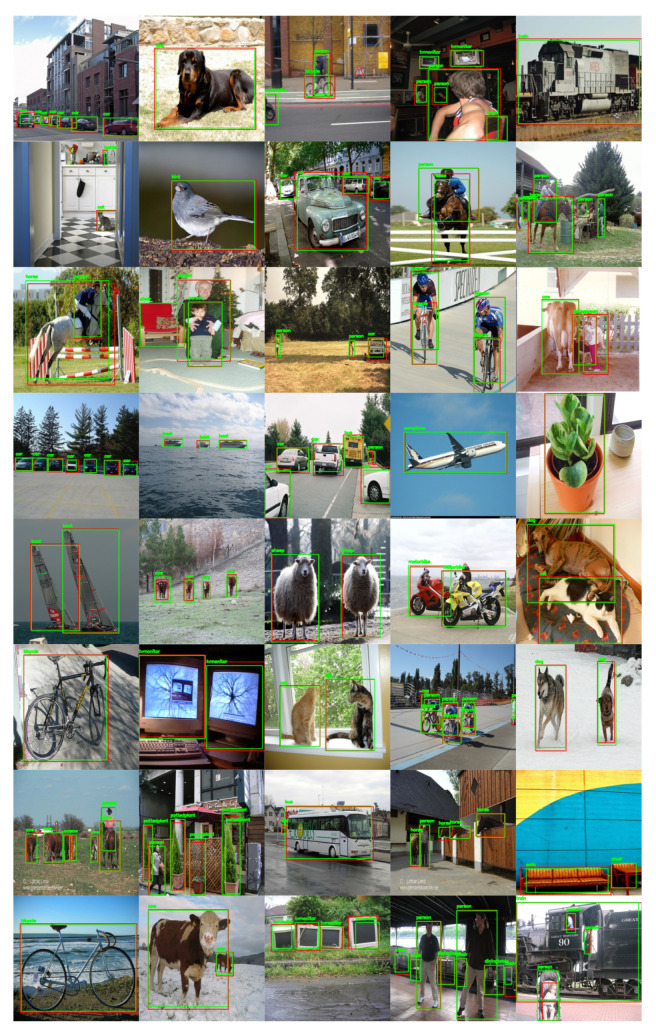
Sample results obtained by the proposed method on random images from PASCAL VOC2007 test set. The green and red bounding boxes refer to the detection and ground truth boxes, respectively.

**Figure 9:**
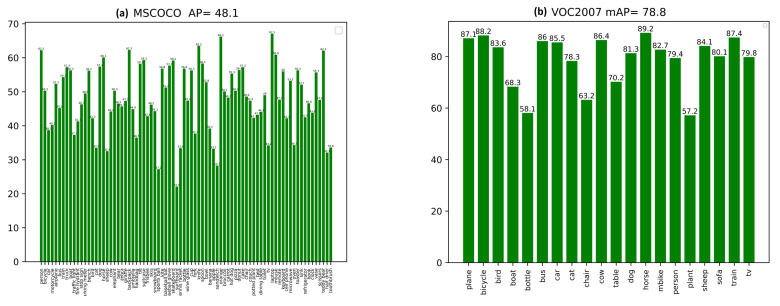
(**a**) The obtained evaluation mAP value of each class of PASCAL VOC2007 test set and the overall mean AP value at IOU of 0.5; (**b**) the AP${}_{box}$ value for each class MS-COCO minival and the overall mean value of AP${}_{box}$.

While training the PASCAL VOC dataset, we combined the training and validation datasets of PASCAL VOC2007 and PASCAL VOC2012 to increase the training data as much as possible. During the testing and performance evaluation, we evaluated the VOC2007 test set. The proposed method could attain an mAP @ IOU of 0.5 values of 78.8 on PASCAL VOC2007. This high mAP is obtained by tuning the best parameters of PPHT so the model can detect both small and large objects. The tuning of PPHT parameters is very sensitive and needs to choose the best combinations of ρ, θ, and *t* as each parameter has a great impact on the final detection results; the parameters selected for each one of the datasets, according to Algorithm 1, are shown in Table 1. While tuning the PPHT parameters, we notice that increasing *t* provides more points for line detection but also has a negative effect when many small boxes are detected. θ is the angular resolution of the accumulator, and when it increases, PPHT can combine very close lines, while ρ, which is the distance resolution of the accumulator, controls the length of the detected line segments. All the parameters should be tuned together to obtain the best detection results. Figure 8 shows the sample results obtained by the proposed method on the PASCAL VOC2007 test set where the results show the ability of the proposed model to detect both small and large objects accurately. The mAP of each class of the two datasets is also reported in Figure 9a. The model could process the frames at a rate of 27 FPS, which is good enough for real-time applications.

### 9.3. Evaluation Results on Ms-COCO Minival

**Figure 10:**
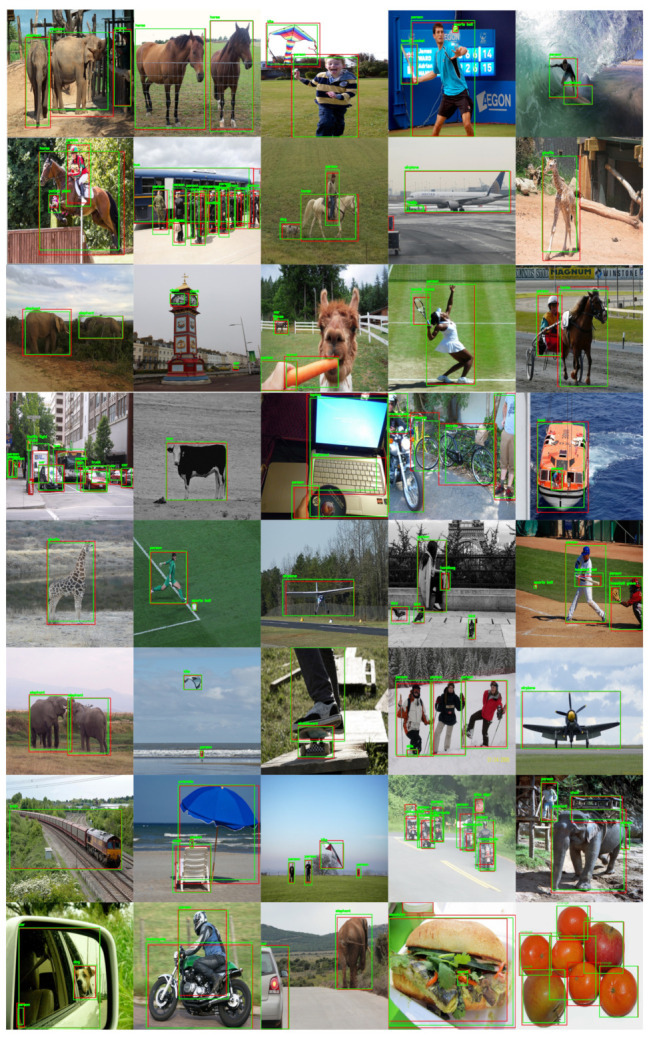
Sample results were obtained by the proposed method on random images from MS-COCO minival dataset. The green and red boxes refer to the detected and ground truth boxes, respectively.

During the evaluation on MS-COCO minival, the proposed method could attain a box average precision (AP${}_{box}$) of 48.1, which is relatively high for a hard dataset such as MS-COCO. The model could produce accurate detections as shown in Figure 10; however, the model struggles with the very crowded scenes of sports matches with a large number of people; such scenes are common in MS-COCO images. We also used the manual tuning of the PPHT parameters to achieve the best detection results; the PPHT parameters are reported in Table 3. Figure 10 shows sample detection results obtained by the proposed model that was trained on the MS-COCO dataset. The AP${}_{box}$ of each class is also shown in Figure 9b. The model could attain a frame rate of 21.3 FPS, which still is an acceptable speed for real-time processing.

### 9.4. Performance Comparison with SOTA Methods

We compared the proposed method (LEOD-Net) with the state-of-the-art (SOTA) methods of object detection. As there are hundreds of object detection methods, we selected the most relevant methods at least in terms of complexity and input image size; we also included a few popular two-stage methods in our comparison to highlight the high accuracy of our method. Regarding our model that was trained on PASCAL VOC2007, it can outperform the other SOTA methods, including the two-stage methods, in terms of AP${}_{box}$, except for YOLOV4, which is a recent method applying multiple techniques to increase the performance. The speed is considered average as it is not as high as YOLOV2 and it is not slow similar to the two-stage methods as reported in Table 4. Regarding our model that was trained on MS-COCO 2017, it ranked as the second-best method (AP${}_{box}=48.11$) after YOLOV4 (AP${}_{box}=50.51$) as reported in Table 5. The evaluation on the COCO minival dataset was performed according to a recent study in [35]. Regarding the speed comparison on the MS-COCO dataset, we did not include the comparison of the speed as each method was tested on a different environment and hardware. In general, the proposed method (LEOD-Net) could attain a notable mAP that can work in real-time, which is the best trade-off for any object detection method.

## 10. Limitations and Future Work

Although we have attained good performance for our model, the model has a weakness in the optimization of PPHT parameters. To address this weakness, we aim to employ an automatic parameter selection using a search method in a future work instead of the manual tuning of the PPHT parameters. We believe that more tuning for the PPHT may produce better object detection results, so automatic tuning can be very helpful since the parameters have cross relations and also depend on other factors such as image size and quality of the estimated features before the pixel-shuffle operation. In addition, we plan to employ vision transformers (ViT) [36] in a future work to exploit the general context learning, which can be achieved using ViT and can be used to generate richer line features. Since the obtained object detection results are so promising, we aim also to extend the method in the future to perform instance segmentation in collaboration with our previous semantic segmentation method proposed in [25], which is one of the most-difficult high-level computer vision tasks. This method uses the original Xception architecture for semantic segmentation, so the same architecture can be trained to perform both object detection and semantic segmentation simultaneously, which is instance segmentation. In addition, the future method could attain real-time processing since our proposed method and the suggested segmentation method can work at a high processing speed.

## 11. Conclusions

We propose an object detection method using line-encoded bounding boxes (LEOD-Net), which proved to be efficient enough for object detection at a high speed (27 fps on PASCAL VOC and 21.3 on MS-COCO) via our experiments. The proposed method exploits the progressive probabilistic Hough transform to refine the initial pseudo-line masks predicted by the proposed CNN model and form the bounding boxes. The parameters of PPHT are so sensitive in the output detection and should be tuned carefully to obtain the best line decoding results. The proposed method outperforms many SOTA methods in terms of accuracy and some methods in terms of frame processing speed. The obtained qualitative results show the high performance of the proposed method in detecting accurate bounding boxes which match the object boundaries. Finally, the mAP values are good enough for accurate object detection tasks.

## Figures and Tables

**Table 1:** Comparison between the different scales of the line mask in terms of mAP and speed on PASCAL VOC2007 with the best selection of $\rho_{1,2,3}$, $\theta_{1,2,3}$ and $t_{1,2,3}$. The best value is shown in bold.

Scale	$\rho_{1,2,3}$	$\theta_{1,2,3}$	$t_{1,2,3}$	mAP	FPS
1/8	1.0, 0.98, 0.96	0.80, 0.90, 1.0	10, 15, 25	53.2	30.1
1/4	0.96, 0.95, 0.94	0.82, 0.92, 0.99	8, 12, 23	**78.8**	25.0
1/2	0.95, 0.94, 0.93	0.85, 0.94, 0.96	5, 10, 22	46.3	21.2
1	0.94, 0.92, 0.90	0.87, 0.96, 0.92	5, 10, 18	33.5	17.3

**Table 2:** The complexity analysis for the proposed architecture calculating the number of floating point operations in billions (B) for the convolution2D (Conv2D) layer, depth-wise separable convolution2D (DWConv2D), maxpooling2D (MP2D), multiplications, and additions. The total number of floating point operations (FLOPs) in billions and the total number of parameters (Params.) in full precision are also shown. The image size of 448×448 and 560×560 are the image sizes for the models trained on PASCAL VOC and MS-COCO, respectively.

Image Size	Conv2D	DWConv2D	MP2D	Mul.	Add.	# FLOPs	# Params.
448×448	36.3500	0.5856	0.0283	0.0210	0.0210	37.0104	21,054,812
560×560	57.0500	0.9178	0.0445	0.0210	0.0210	58.0528	21,054,812

**Table 3:** The best PPHT parameters obtained during tuning for each dataset, mAP${}_{0.5}$, and FPS for the proposed model tested on the VOC2007 test set in addition to AP${}_{box}$, AP${}_{0.5,0.75,0.95}$, on MS-COCO minival using the same terms.

Data Set	$\rho_{1,2,3}$	$\theta_{1,2,3}$	$t_{1,2,3}$	mAP${}_{0.5}$	AP${}_{\mathrm{box}}$	AP${}_{S/M/L}$	FPS
VOC2007	0.96, 0.95, 0.94	0.82, 0.92, 0.99	8, 12, 23	78.8	-	-	27.0
MS-COCO	0.96, 0.95, 0.93	0.82, 0.92, 1.00	5, 12, 22	-	48.1	19.24/45.33/57.35	21.3

**Table 4:** Comparison with SOTA methods on the VOC2007 test set while training the model on VOC2007+VOC2012 trainval datasets together. The best value of AP${}_{box}$ is shown in bold. The second best value is shown with underline.

Method	Backbone	Input Size	mAP${}_{0.5}$	FPS	GPU
RCNN [1]	AlexNet	500×500	66.0	0.02	Tesla K20
Fast RCNN [2]	VGG16	1000×600	70.0	0.5	Titan X
Faster RCNN [3]	VGG16	1000×600	73.3	7.0	Titan XP
Faster R-CNN [3]	ResNet101	1000×600	76.4	5.0	Titan XP
SSD300 [9]	VGG16	300×300	74.3	46.0	Titan X
SSD512 [9]	VGG16	512×512	76.8	19.0	Titan X
YOLO [5]	Darknet	416×416	63.4	45.0	Titan X
YOLO [5]	VGG-16	416×416	66.4	45.0	Titan X
YOLOV2 [6]	Darknet-19	416×416	76.8	67.0	Titan X
YOLOV2 [6]	Darknet-19	480×480	77.8	59.0	Titan X
YOLOV2 [6]	Darknet-19	512×512	78.6	40.0	Titan X
YOLOV3 [7]	Darknet-53	544×544	78.6	40.0	Titan X
YOLOV4 [8]	EEEA-Net	416×416	**81.8**	43.0	RTX2080-Ti
**LEOD-Net (ours)**	Xception-16	448×448	78.8	27.0	Titan XP

**Table 5:** Comparison between the proposed method (LEOD-NET) and SOTA methods on COCO val2017 (COCO minival) while training the model on COCO train2017 dataset. The best value of AP${}_{box}$ is shown in bold. The second best value is shown with underline.

Method	Backbone	Input Size	AP${}_{\mathrm{box}}$	AP${}_{0.5/0.75}$	AP${}_{S/M/L}$
Faster RCNN [3]	ResNet101	1000×600	42.42	49.06/39.04	17.06/38.59/48.15
Faster RCNN [3]	ResNet50	1000×600	40.52	47.39/37.27	15.32/36.52/46.14
RetinaNet-FPN [10]	ResNet101	1000×600	40.41	44.15/35.67	13.92/35.88/44.42
RetinaNet-FPN [10]	ResNet50	1000×600	38.69	44.32/35.20	14.11/34.80/44.270
SSD640 [9]	ResNet101	640×640	35.60	39.60/31.83	8.28/30.93/45.02
SSD640 [9]	ResNet50	640×640	34.19	37.92/30.25	8.22/28.63/43.29
YOLOV2 [6]	DarkNet19	416×416	29.39	24.41/4.98	42.13/25.69/6.44
YOLOv2-tiny [6]	DarkNet19	416×416	10.53	17.75/5.52	0.67/6.75/15.55
YOLOV3 [7]	DarkNet53	640×640	38.84	48.99/33.48	16.69/33.11/42.04
YOLOv3-spp [7]	DarkNet53	640×640	42.59	49.47/38.02	16.96/34.60/48.09
YOLOv4 [8]	CSPDarknet-53	512×512	**50.50**	54.63/46.24	23.10/46.01/53.27
YOLOv4-tiny [8]	CSPDarknet-53	416×416	21.97	28.64/ 17.23	4.80/19.91/24.84
CenterNet [21]	Hourglass104	512×512	44.47	44.61/38.29	19.80/36.08/44.60
EfficientDet [22]	EfficientNet-B1	640×640	39.18	41.75/34.64	8.08/35.40/50.42
EfficientDet [22]	EfficientNet-B0	512×512	33.48	39.02/30.90	6.00/31.04/47.84
**LEOD-Net (ours)**	Xception-16	560×560	48.11	53.21/44.33	19.24/45.33/57.35

## Data Availability

The datasets used in this paper are public datasets.
